# Serological Evidence of *Orthopoxvirus* Infection in Neotropical Primates in Brazil

**DOI:** 10.3390/pathogens11101167

**Published:** 2022-10-10

**Authors:** Filipe Vieira Santos de Abreu, Kamila Lorene Soares Rocha, Ramon Silva-Oliveira, Mariana Viana Macedo, Thamires Gabriele Macedo Silva, Maria Eduarda Gonçalves-dos-Santos, Cirilo Henrique de Oliveira, Sandy Micaele Aquino-Teixeira, Vinícius de Oliveira Ottone, Alex Junio Jardim da Silva, Ronaldo Medeiros dos Santos, Aline Tátila-Ferreira, Marco Antônio Barreto de Almeida, Edmilson dos Santos, Jáder da Cruz Cardoso, Aline Alves Scarpellini Campos, George Rego Albuquerque, Anaiá da Paixão Sevá, Bergmann Morais Ribeiro, Danilo Simonini Teixeira, Fabrício Souza Campos, Ana Cláudia Franco, Paulo Michel Roehe, Giliane de Souza Trindade, Danilo Bretas de Oliveira

**Affiliations:** 1 Insect Behavior Laboratory, Instituto Federal do Norte de Minas Gerais, Salinas 39560-000, Minas Gerais, Brazil; 2 Laboratório de Vírus, Departamento de Microbiologia, Instituto de Ciências Biológicas, Universidade Federal de Minas Gerais, Belo Horizonte 31270-901, Minas Gerais, Brazil; 3 Medical School, Health Science Post-Graduate Program, Universidade Federal dos Vales do Jequitinhonha e Mucuri, Diamantina 39100-000, Minas Gerais, Brazil; 4 Centro Estadual de Vigilância em Saúde, Secretaria de Saúde do Rio Grande do Sul, Porto Alegre 90450-190, Rio Grande do Sul, Brazil; 5 Department of Agricultural and Environmental Sciences, State University of Santa Cruz, Ilhéus 45662-900, Bahia, Brazil; 6 Cell Biology Department, Biology Institute, Universidade de Brasília, Brasília 70910-000, Brazil; 7 Laboratory of Bioinformatics and Biotechnology, Universidade Federal de Tocantins, Gurupi 77402-970, Tocantins, Brazil; 8 Laboratório de Virologia—Departamento de Microbiologia, Imunologia e Parasitologia, Universidade Federal do Rio Grande do Sul, Porto Alegre 90050-170, Rio Grande do Sul, Brazil

**Keywords:** *Poxviridae*, non-human primates, vaccinia virus, plaque reduction neutralization test

## Abstract

The genus *Orthopoxvirus* (OPXV) of the family *Poxviridae* comprises several viruses that are capable of infecting a wide range of hosts. One of the most widespread OPXVs is the Vaccinia virus (VACV), which circulates in zoonotic cycles in South America, especially in Brazil, infecting domestic and wild animals and humans and causing economic losses as well as impacting public health. Despite this, little is known about the presence and/or exposure of neotropical primates to orthopoxviruses in the country. In this study, we report the results of a search for evidence of OPVX infections in neotropical free-living primates in the state of Minas Gerais, southeast Brazil. The sera or liver tissues of 63 neotropical primates were examined through plaque reduction neutralization tests (PRNT) and real-time PCR. OPXV-specific neutralizing antibodies were detected in two sera (4.5%) from *Callithrix penicillata*, showing 55% and 85% reduction in plaque counts, evidencing their previous exposure to the virus. Both individuals were collected in urban areas. All real-time PCR assays were negative. This is the first time that evidence of OPXV exposure has been detected in *C. penicillata*, a species that usually lives at the interface between cities and forests, increasing risks of zoonotic transmissions through spillover/spillback events. In this way, studies on the circulation of OPXV in neotropical free-living primates are necessary, especially now, with the monkeypox virus being detected in new regions of the planet.

## 1. Introduction

The genus *Orthopoxvirus* (OPXV) of sub-family *Chordopoxvirinae*, family *Poxviridae*, comprises several viruses that are capable of infecting a wide range of hosts. The viruses belonging to this genus are highly complex and share several similarities. This is epidemiologically important since the OPXVs can confer cross-immunity to each other. One of the most widespread OPXVs is the vaccinia virus (VACV), the prototype of the genus *Orthopoxvirus*, which circulates in zoonotic cycles and infects the cattle and workers in rural areas of Brazil, causing economic losses and impacting public health [1,2]. 

Since the beginning of the 21st century, VACV has been detected throughout Brazilian territories and has also been found in free-living animals such as rodents, marsupials, procyonids and non-human primates in the Amazon region [3,4], which has increased concerns about wildlife health and sporadic human spillovers, as illustrated by the recent monkeypox outbreak [5,6]. Despite this, little is known about the presence and/or exposure of neotropical primates to the VACV in other regions of Brazil. Therefore, this is the first report of a sampling effort to detect serological and virological evidence of VACV/OPXV infections in neotropical free-living primates in Minas Gerais, which is a region in Brazil that is considered to be the epicenter of VACV outbreaks involving livestock and humans [7,8].

## 2. Materials and Methods

### 2.1. Sampling Effort

**Figure 1:**
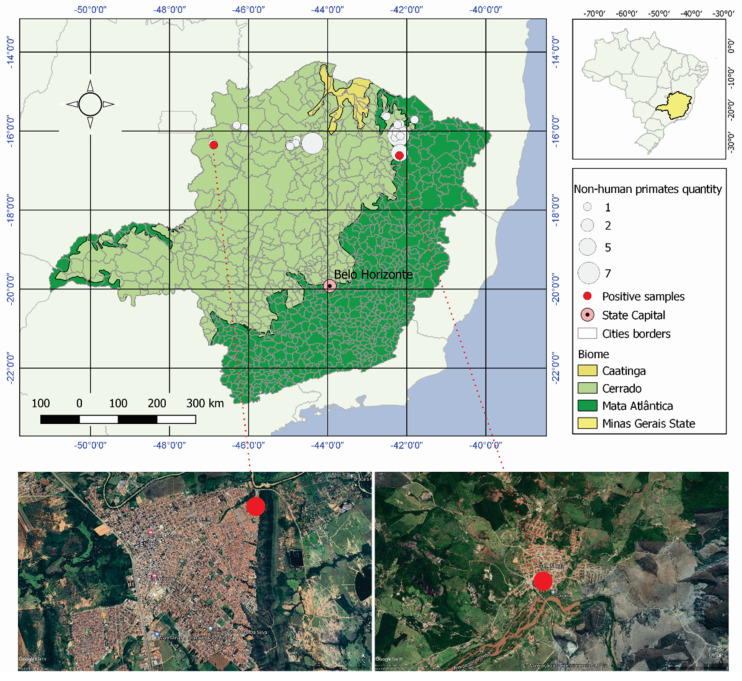
Map showing sampling points and biomes of Minas Gerais, Brazil. The two sera where OPXV-specific neutralizing antibodies were detected are shown with red dots. Satellite images show points of collection of OPXV antibody-positive NHPs in the urban areas of Unaí (**left**) and Coronel Murta (**right**). The figure was created using QGIS software version 3.10 and Google Earth.

Samples from neotropical primates were collected between July 2020 and January 2022 from 13 municipalities spread across the northern region of Minas Gerais, Brazil (Figure 1, Table 1). The area is predominantly within the Cerrado biome (a Savannah-like environment) but presents ecotones between the Caatinga and the Atlantic Forest (Figure 1). Sampling points varied in each municipality, covering urban, rural or sylvatic areas (Table 1). Free-living marmosets were captured using Tomahawk automatic traps and were examined as described elsewhere [9]. Sick or dead marmosets and howler monkeys collected through a previously established information network were also examined [10,11]. Serum and liver tissues samples were collected and frozen in liquid nitrogen (−196 °C) until the performance of serological and molecular assays. All protocols were previously approved by the Institutional Ethics Committee for Animal Experimentation (Protocol CEUA/IFNMG n° 14/2019) and by the Brazilian Ministry of the Environment (SISBIO n° 71714-2).

### 2.2. Plaque Reduction Neutralization Test (PRNT)

To assess the presence of OPXV-neutralizing antibodies, we used a plaque reduction neutralization test (PRNT), which is considered the gold standard for the differential diagnosis of OPXV antibodies. Such an assay has shown reliability, high specificity, and has been used in a number of seroprevalence studies that were designed to detect anti-OPXV neutralizing antibodies in different animal species [3,4,12]. The PRNT was performed as previously reported [13]. Essentially, serum was inactivated at 56 °C for 30 min and then diluted 1:20 in Eagle’s Minimum Essential Medium (MEM) (GIBCO^®^, Whaltam, USA) free of fetal bovine serum (FBS). The samples were mixed with an equal volume of a virus suspension containing approximately 150 plaque-forming units (PFU) of VACV strain Western Reserve. The solution was homogenized and incubated for 16 h at 37 °C, in a 5% CO_2_ atmosphere. Six-well plates containing BSC40 cells monolayers (CRL-2761, ATCC^®^, Manassas, USA) at 80% confluence were inoculated with virus/serum mix solutions and incubated at 37 °C for 1 h in 5% CO_2_ atmosphere. Subsequently, MEM with 2% FBS was added to each well and incubated for 2 days at 37 °C in a 5% CO_2_ atmosphere. When typical VACV-WR cytopathic effects were clearly observed, all monolayers were fixed with 3.7% formaldehyde and stained with 1% crystal violet (SYNTH^®^, Diadema, Brazil). Controls with infected and uninfected cells were included in each plate. To maintain the viability of the virus control, fetal bovine serum (FBS) was added to this solution at the same concentration (2.5%). The cell control contained 2.0% FBS media only. All samples were tested in triplicate. Aiming to guarantee high specificity, a serum sample was considered positive when an equal to or greater than 50% reduction in PFUs was detected, when compared to virus controls.

### 2.3. Real-Time PCR Assays

In order to improve the sensitivity and specificity of the real-time PCR serum, liver tissues were tested through two singleplex assays targeting two different OPXV genes: the C11R gene, related to the virus growth factor (VGF), a usually duplicated and conserved gene; and the A56R gene, which codes the viral hemagglutinin (HA) and is an important marker for molecular diagnostics. The primer sequences utilized were C11R F (5′ CGCTACAACAGATATTCCAGCTATCAG 3′), C11R R (5′ AGCGTGGATACAGTCACCGTGTAA 3′), A56 F (5′ CATCATCTGGAATTGTCACTACTAAA 3′), A56 R (5′ ACGGCCGACAATATAATTAATGC 3′) [3,14]. The two targets were tested in duplicate in a final volume of 10 μL in a StepOne^®^ (Applied Biosystems, Foster City, USA) apparatus. The C11R and A56R genes were tested using SYBR^®^ Green I Master Mix with the following settings: a cycle of DNA denaturation at 95 °C/20 min, 40 cycles of 95 °C/3 s and 60 °C/20 s, and a melting curve using 95 °C/3 s and 60 °C/20 s, followed by 4 °C increases in temperature up to 95 °C/15 s.

## 3. Results

The sampling efforts resulted in the collection of tissues from 63 neotropical primates belonging to three species (*Callithrix penicillata* and *C. geoffroy*—Callitrichidae family; and *Alouatta caraya*—Atelidae family), which were examined (Table 1, Figure 1). No skin lesions or other clinical signals were found in any of the animals examined. OPXV-specific neutralizing antibodies (more than 50% of neutralization) were detected in two (4.5%) of the 44 tested sera, both from *C. penicillate* (MG39 and MG45 samples, showing 55% and 85% PFU reduction, respectively), evidencing their previous exposure to the virus. Both individuals were sampled in urban areas (Table 1, Figure 1). Their sampling points were 505 km apart in a straight line. All real-time PCR assays in the search for OPXV genomes were negative.

## 4. Discussion

The close relationship between humans and other animals has been increasing due to the growth of the world population, as well as deforestation for food production and animal husbandry, making zoonoses increasingly frequent. These changes directly affect wildlife and bring humans ever closer to pathogens that, thus far, have only circulated in animals, and vice versa, increasing the risks of spillovers and spillbacks [15]. Furthermore, the increasing transport of live animals for trade (eventually involving legal and illegal wildlife specimens), industrialization processes, and global trade lead to an equally large increase in the potential for the emergence and spread of pathogens [16]. Therefore, it is estimated that more than 70% of human infections have a zoonotic origin [17].

The OPXV antibody-positive free-living neotropical primates detected in the current study demonstrate this scenario, since they were all captured in the urban environment, showing that they can live between forest environments and cities, potentially enabling spillover as well as spillback virus events [1,2,3,4]. Several viruses have been shown to be transmitted through such routes, including yellow fever virus and herpes viruses [18,19], even though little is known about several of them in circulation, as is the case with orthopoxviruses such as VACV, originally described in cows, in Brazil [20].

Recently, it has been shown that VACV persists not only in livestock, but also in wild reservoirs (including rodents and other mammals), as well as in equids, captive and domestic animals including cats and dogs [3,4,21]. In the Amazon biome, during a wildlife rescue for the construction of a hydroelectric plant, many wild animals were tested, and non-human primates of the genera *Cebus* and *Alouatta* (family—Cebidae and Atelidae, respectively) showed the highest detection rates of VACV. The animals were captured in a wild area and had no evidence of previous contact with humans and/or dairy cattle [3]. Here, despite a lower seropositivity rate, we demonstrated previous exposure to the OPXV of a new genus and family (*Callithrix*/Callitrichidae) of a neotropical primate living in close contact with humans. Genetics and ecological features could explain the difference that was found. Other studies outside the Amazon have demonstrated the participation of synanthropic and wild rodents or other mammals in the maintenance of the OPXV circulation [22]. An example of this is the VACV infections of domestic dogs and wild coatis (*Nasua nasua*) living in close contact in an overlapping area of urban and wild environments, suggesting a transmission cycle between domestic and wild animals [4,23]. Some rodent species can also function as intermediary hosts, acting as “bridges” between wild animals, domestic animals and humans [22,24]. In this study, samples were tested trough PRNT for serological screening because it is considered the gold standard, and due to the absence of specific reagents for the standardization of an ELISA test [12,25]. However, we are aware of the advantages that ELISA tests provide to serological studies, such as increasing specificity and the possibility of rapid execution [25,26].

Since all of the real-time PCR assays were negative and because serology can show any cross-reaction between different OPXVs, it is not possible to determine whether previous exposures were caused by VACV. However, VACV is the most widespread OPXV in Brazil and is endemic in Minas Gerais. Interestingly, this is the first time that evidence of VACV/OPXV exposition has been detected in *C. penicillata*, a species with a population of more than 10,000 individuals and that can live at the interface between cities and forests (ecotone), being commonly found in several Brazilian urban areas, in close contact with humans [27,28]. Furthermore, the geographic range of the two antibody-positive cases (505 km apart) suggests that this VACV/OPXV–*Callithrix* interaction is wide-ranging in the territory. Thus, studies on the circulation of OPXV in neotropical free-living primates are necessary, especially now, as monkeypox has been introduced to new regions of the planet, creating the possibility of establishing a zoonotic cycle through the occurrence of spillovers and spillbacks. An experimental pathogenesis study has, indeed, demonstrated that a neotropical primate (*Callithrix jacchus*—marmosets) can be infected by low doses of monkeypox virus and can produce a high viremia, as well as pathological signals that are consistent with monkeypox in humans [29]. Thus, VACV could pose a potential risk to public health in the same way as another virus (monkeypox) belonging to the same genus [6].

## Figures and Tables

**Table 1:** Description of samples tested by species, habitat, date of collection, sampling point, tissue and city. Sera were tested through PRNT and real-time PCR. Liver tissues were tested through real-time PCR. OPXV antibody-positive samples are highlighted. Legend: neg = negative; PRNT-pos = positive in the PRNT assays; “−” = tissue not available.

ID	Species	Habitat	Collection Date	Latitude	Longitude	Serum	Liver	City
MG10	*C. penicillata*	Sylvatic	04/07/2020	−16.120847	−42.209519	neg	−	Salinas
MG11	*C. penicillata*	Sylvatic	04/07/2020	−16.120847	−42.209519	neg	−	
MG12	*C. penicillata*	Sylvatic	04/07/2020	−16.120847	−42.209519	neg	−	
MG13	*C. penicillata*	Sylvatic	04/07/2020	−16.120847	−42.209519	neg	−	
MG25	*C. penicillata*	Urban	19/09/2020	−16.160950	−42.293317	neg	−	
MG26	*C. penicillata*	Urban	21/09/2020	−16.160950	−42.293317	neg	−	
MG62	*C. penicillata*	Sylvatic	13/04/2021	−16.026000	−42.266000	−	neg	
MG63	*C. penicillata*	Rural	13/04/2021	−16.157528	−42.311306	−	neg	
MG70	*C. penicillata*	Rural	10/09/2021	−16.157528	−42.311306	neg	−	
MG64	*C. penicillata*	Rural	24/07/2021	−16.157528	−42.311306	−	neg	
MG96	*C. penicillata*	Rural	17/02/2022	−16.15637	−42.30730	−	neg	
MG14	*C. penicillata*	Rural	30/07/2020	−15.711878	−41.800169	neg	−	Berizal
MG15	*C. penicillata*	Rural	30/07/2020	−15.711878	−41.800169	neg	−	
MG32	*C. geoffroyi*	Rural	18/10/2020	−16.12522	−42.159269	neg	−	Araçuaí
MG33	*C. penicillata*	Rural	19/10/2020	−16.553161	−42.176839	neg	−	Coronel Murta
MG34	*C. penicillata*	Rural	19/10/2020	−16.553161	−42.176839	neg	−	
MG35	*C. penicillata*	Rural	20/10/2020	−16.553161	−42.176839	neg	−	
MG36	*C. penicillata*	Rural	20/10/2020	−16.553161	−42.176839	neg	−	
MG38	*C. penicillata*	Rural	20/10/2020	−16.553161	−42.176839	neg	−	
MG39	*C. penicillata*	Urban	21/10/2020	−16.619644	−42.183942	PRNT-POS	−	
MG43	*C. penicillata*	Urban	11/01/2021	−16.352694	−46.881139	neg	−	Unaí
MG45	*C. penicillata*	Urban	11/01/2021	−16.352694	−46.881139	PRNT-POS	−	
MG46	*C. penicillata*	Urban	11/01/2021	−16.352694	−46.881139	neg	−	
MG48	*A. caraya*	Rural	17/01/2021	−16.308444	−46.907722	neg	−	
MG49	*A. caraya*	Rural	17/01/2021	−16.308445	−46.907723	neg	−	
MG50	*C. penicillata*	Rural	19/01/2021	−15.911472	−46.099972	neg	−	Arinos
MG51	*C. penicillata*	Rural	19/01/2021	−15.848770	−46.300809	neg	−	
MG52	*C. penicillata*	Urban	20/03/2021	−15.609222	−42.542694	neg	−	Rio Pardo de Minas
MG53	*C. penicillata*	Urban	20/03/2021	−15.609222	−42.542694	neg	−	
MG54	*C. penicillata*	Rural	20/03/2021	−15.629972	−42.508472	neg	−	
MG55	*C. penicillata*	Rural	20/03/2021	−15.629972	−42.508472	neg	−	
MG56	*C. penicillata*	Urban	22/03/2021	−15.807389	−42.239111	neg	−	Taiobeiras
MG57	*C. penicillata*	Urban	22/03/2021	−15.807389	−42.239111	neg	−	
MG58	*C. penicillata*	Rural	23/03/2021	−15.817889	−42.159972	neg	−	
MG59	*C. penicillata*	Rural	23/03/2021	−15.817889	−42.159972	neg	−	
MG60	*C. penicillata*	Sylvatic	24/03/2021	−15.841139	−42.229750	neg	−	
MG61	*C. penicillata*	Sylvatic	24/03/2021	−15.841139	−42.229750	neg	−	
MG66	*A. caraya*	Sylvatic	25/08/2021	−16.217389	−44.783694	−	neg	Icaraí de Minas
MG72	*A. caraya*	Sylvatic	13/09/2021	16.340278	−44.947139	−	neg	
MG73	*A. caraya*	Sylvatic	13/09/2021	−16.356083	−44.965333	−	neg	
MG74	*A. caraya*	Sylvatic	13/09/2021	−16.356083	−44.965333	−	neg	
MG68	*C. penicillata*	Rural	26/08/2021	−16.311667	−44.810000	−	neg	Ubaí
MG76	*A. caraya*	Sylvatic	16/09/2021	−16.385444	−44.947083	−	neg	
MG78	*C. penicillata*	Rural	17/01/2022	−15.44731	−44.37050	neg	−	Januária
MG79	*C. penicillata*	Rural	17/01/2022	−15.44731	−44.37050	neg	−	
MG90	*C. penicillata*	Rural	20/01/2022	−15.44731	−44.37050	neg	−	
MG91	*C. penicillata*	Rural	23/01/2022	−15.44731	−44.37050	neg	−	
MG75	*C. penicillata*	Rural	14/09/2021	−16.354417	−44.349639	−	neg	Brasília de Minas
MG77	*A. caraya*	Sylvatic	20/11/2021	−16.32207	−44.42859	−	neg	
MG80	*A. caraya*	Sylvatic	18/01/2022	−16.309691	−44.382729	−	neg	
MG81	*A. caraya*	Sylvatic	18/01/2022	−16.309644	−44.382161	−	neg	
MG82	*A. caraya*	Sylvatic	17/01/2022	−16.309691	−44.382729	−	neg	
MG83	*A. caraya*	Sylvatic	17/01/2022	−16.309691	−44.382729	−	neg	
MG84	*A. caraya*	Sylvatic	17/01/2022	−16.309694	−44.382709	−	neg	
MG85	*A. caraya*	Sylvatic	17/01/2022	−16.309664	−44.382386	−	neg	
MG86	*C. penicillata*	Rural	18/01/2022	−16.30962	−44.38238	neg	−	
MG87	*C. penicillata*	Rural	18/01/2022	−16.30962	−44.38238	neg	−	
MG88	*C. penicillata*	Rural	18/01/2022	−16.30962	−44.38238	neg	−	
MG89	*A. caraya*	Sylvatic	19/01/2022	−16.306517	−44.383528	−	neg	
MG92	*C. penicillata*	Sylvatic	25/02/2022	−15.348677	−44.900128	neg	−	Bonito de Minas
MG93	*C. penicillata*	Sylvatic	25/02/2022	−15.348677	−44.900128	neg	−	
MG94	*C. penicillata*	Rural	29/01/2022	−15.346668	−44.676110	neg	−	
MG95	*C. penicillata*	Rural	29/01/2022	−15.346668	−44.676110	neg	−	

## Data Availability

Not applicable.

## References

[1] Kroon E.G., Mota B.E.F., Abrahão J.S., da Fonseca F.G., Trindade G.D.S. (2011). Zoonotic Brazilian Vaccinia virus: From field to therapy. Antivir. Res..

[2] de Oliveira J.S., Figueiredo P.D.O., Costa G.B., de Assis F.L., Drumond B.P., Da Fonseca F.G., Nogueira M.L., Kroon E.G., Trindade G.D.S. (2017). Vaccinia virus natural infections in Brazil: The good, the bad, and the ugly. Viruses.

[3] Abrahão J.S., Silva-Fernandes A.T., Lima L.S., Campos R.K., Guedes M.I., Cota M.M., Assis F.L., Borges I.A., Souza-Júnior M.F., Lobato Z.I. (2010). Vaccinia virus infection in monkeys, Brazilian Amazon. Emerg. Infect. Dis..

[4] Costa G.B., De Almeida L.R., Cerqueira A.G.R., Mesquita W.U., De Oliveira J.S., Miranda J.B., Saraiva-Silva A.T., Abrahão J.S., Drumond B.P., Kroon E.G. (2018). Vaccinia virus among domestic dogs and wild coatis, Brazil, 2013–2015. Emerg. Infect. Dis..

[5] Bunge E.M., Hoet B., Chen L., Lienert F., Weidenthaler H., Baer L.R., Steffen R. (2022). The changing epidemiology of human mon-keypox—A potential threat? A systematic review. PLoS Negl. Trop. Dis..

[6] WHO (2022). Multi-Country Monkeypox Outbreak in Non-Endemic Countries. https://www.who.int/emergencies/disease-outbreak-news/item/2022-DON385.

[7] Leite J.A., Drumond B.P., Trindade G.S., Lobato Z.I.P., Da Fonseca F.G., Dos Santos J.R., Madureira M.C., Guedes M.I.M.C., Ferreira J.M.S., Bonjardim C.A. (2005). Passatempo virus, a vaccinia virus strain, Brazil. Emerg. Infect. Dis..

[8] Trindade G.S., Lobato Z.I.P., Drumond B.P., Leite J.A., Trigueiro R.C., Guedes M.I.M.C., Da Fonseca F.G., dos Santos J.R., Bonjardim C.A., Ferreira P.C.P. (2006). Short report: Isolation of two Vaccinia virus strains from a single bovine vaccinia outbreak in rural area from Brazil: Implications on the emergence of zo-onotic orthopoxviruses. Am. J. Trop. Med. Hyg..

[9] de Abreu F.V.S., Ferreira-De-Brito A., Azevedo A.D.S., Linhares J.H.R., Santos V.D.O., Miranda E.H., Neves M.S.A.S., Yousfi L., Ribeiro I.P., dos Santos A.A.C. (2020). Survey on Non-Human Primates and Mosquitoes Does not Provide Evidences of Spillover/Spillback between the Urban and Sylvatic Cycles of Yellow Fever and Zika Viruses Following Severe Outbreaks in Southeast Brazil. Viruses.

[10] Abreu F.V.S., dos Santos E., Gomes M.Q., Vargas W.P., Oliveira Passos P.H., Nunes e Silva C., Araújo P.C., Pires J.R., Romano A.P.M., Teixeira D.S. (2019). Capture of *Alouatta guariba clamitans* for the surveillance of sylvatic yellow fever and zoonotic malaria: Which is the best strategy in the tropical Atlantic Forest?. Am. J. Primatol..

[11] Andrade M.S., Campos F.S., Oliveira C.H., de Oliveira R.S., Campos A.A.S., Almeida M.A.B., Simonini-Teixeira D., da Sevá A.P., Temponi A.O.D., Magalhães F.M. (2021). Fast surveillance response and genome sequencing reveal the circulation of a new Yellow Fever Virus sublineage in 2021, in Minas Gerais, Brazil. bioRxiv..

[12] Kroon G.E., Santos Abrahão J., de Souza Trindade G., Pereira Oliveira G., Moreira Franco Luiz A.P., Barbosa Costa G., Lima M.T., Calixto R.S., de Oliveira D.B., Drumond B.P. (2016). Natural vaccinia virus infection: Diagnosis, isolation, and characterization. Curr. Protoc. Microbiol..

[13] Newman F.K., Frey S.E., Blevins T.P., Mandava M., Bonifacio A., Yan L., Belshe R.B. (2003). Improved assay to detect neu-tralizing antibody following vaccination with diluted or undiluted vaccinia (Dryvax) vaccine. J. Clin. Microbiol..

[14] Trindade G.S., Emerson G.L., Carroll D.S., Kroon E., Damon I.K. (2007). Brazilian vaccinia viruses and their origins. Emerg. Infect. Dis..

[15] Jenkins E., Simon A., Bachand N., Stephen C. (2015). Wildlife parasites in a One Health world. Trends Parasitol..

[16] Green S. (2022). The Bioeconomics of Domesticating Zoonoses. Cult. Anthr..

[17] Jones K.E., Patel N.G., Levy M.A., Storeygard A., Balk D., Gittleman J.L., Daszak P. (2008). Global trends in emerging infectious diseases. Nature.

[18] Longa C.S., Bruno S.F., Pires A.R., Romijn P.C., Kimura L.S., Costa C.H.C. (2011). Human Herpesvirus 1 in wild marmosets, Brazil, 2008. Emerg. Infect. Dis..

[19] Mares-Guia M.A.M.D.M., Horta M.A., Romano A., Rodrigues C.D.S., Mendonça M.C.L., Dos Santos C.C., Torres M.C., Araujo E.S.M., Fabri A., De Souza E.R. (2020). Yellow fever epizootics in non-human primates, Southeast and Northeast Brazil (2017 and 2018). Parasites Vectors.

[20] Domingos J.S.I., Silva de Oliveira J., Rocha L.S.R.K., Oliveira D.B., Kroon G.E., Barbosa G.C., Trindade G.S. (2021). Twenty Years after Bovine Vaccinia in Brazil: Where We Are and Where Are We Going?. Pathogens.

[21] Peres M.G., Bacchiega T.S., Appolinário C.M., Vicente A.F., Allendorf S.D., Antunes J.M.A.P., Moreira S.A., Legatti E., Fonseca C.R., Pituco E.M. (2013). Serological study of vaccinia virus reservoirs in areas with and without official reports of outbreaks in cattle and humans in São Paulo, Brazil. Arch. Virol..

[22] Abrahão J.S., Guedes M.I., Trindade G.S., Fonseca F.G., Campos R.K., Mota B.F., Lobato Z.I.P., Silva-Fernandes A.T., Rodrigues G.O.L., Lima L.S. (2009). One more piece in the VACV ecological puzzle: Could peridomestic rodents be the link between wildlife and bovine vaccinia outbreaks in Brazil?. PLoS ONE.

[23] Peres M.G., Bacchiega T.S., Appolinário C.M., Vicente A.F., Mioni M.S.R., Ribeiro B.L.D., Fonseca C.R.S., Pelícia V.C., Ferreira F., Abrahão J.S. (2018). Vaccinia virus in feces and urine of wild rodents from São Paulo State, Brazil. Viruses.

[24] Miranda J.B., Borges I.A., Campos S.P.S., Vieira F.N., De Ázara T.M.F., Marques F.A., Costa G.B., Luis A.P.M.F., De Oliveira J.S., Ferreira P.C.P. (2017). Serologic and molecular evidence of vaccinia virus circulation among small mammals from different biomes, Brazil. Emerg. Infect. Dis..

[25] Cohen B., Doblas D., Andrews N. (2008). Comparison of plaque reduction neutralisation test (PRNT) and measles virus-specific IgG ELISA for assessing immunogenicity of measles vaccination. Vaccine.

[26] Gallardo-Romero N.F., Arechiga-Ceballos N., Emerson G.L., Martínez-Martínez F.O., Doty J.B., Nakazawa Y.J., Rendón-Franco E., Muñoz-García C.I., Villanueva-García C., Ramírez-Cid C. (2016). Endemic Orthopoxvirus circulating in procyonids in Mexico. J. Wildl. Dis..

[27] Teixeira B., Hirsch A., Goulart V.D.L.R., Passos L.P., Teixeira C.P., James P., Young R.J. (2015). Good neighbours: Distribution of black-tufted marmoset (*Callithrix penicillata*) in an urban environment. Wildl. Res..

[28] Bicca-Marques J., Jerusalinsky L., Mittermeier R.A., Pereira D., Ruiz-Miranda C., Rímoli J., Valença Montenegro M., do Valle R.R. (2018). Callithrix penicillata. The IUCN Red List of Threatened Species 2018: E. T41519A1793579.

[29] Mucker E.M., Chapman J., Huzella L.M., Huggins J.W., Shamblin J., Robinson C.G., Hensley L.E. (2015). Susceptibility of mar-mosets (*Callithrix jacchus*) to monkeypox virus: A low dose prospective model for monkeypox and smallpox disease. PLoS ONE.

